# Advancing precision oncology through systematic germline and tumor genetic analysis: The oncogenetic point of view on findings from a prospective multicenter clinical trial of 666 patients

**DOI:** 10.1002/cam4.6498

**Published:** 2023-09-11

**Authors:** Benoit Mazel, Geoffrey Bertolone, Amandine Baurand, Elodie Cosset, Caroline Sawka, Marion Robert, Elodie Gautier, Allan Lançon, Manon Réda, Laure Favier, Valentin Dérangère, Corentin Richard, Christine Binquet, Romain Boidot, Vincent Goussot, Juliette Albuisson, François Ghiringhelli, Laurence Faivre, Sophie Nambot

**Affiliations:** ^1^ Centre de Génétique, FHU‐TRANSLAD, Centre Hospitalier Universitaire Dijon‐Bourgogne Dijon France; ^2^ INSERM UMR 1231 GAD, Génétique des Anomalies du Développement, Université Bourgogne Franche‐Comté Dijon France; ^3^ Unité d'Oncogénétique, Centre de Lutte Contre le Cancer Georges François Leclerc—UNICANCER Dijon France; ^4^ Département d'Oncologie Médicale Centre de Lutte Contre le Cancer Georges François Leclerc—UNICANCER Dijon France; ^5^ Plateforme de Transfert en Biologie Cancérologique Centre de Lutte Contre le Cancer Georges François Leclerc—UNICANCER Dijon France; ^6^ INSERM UMR 1231 GIMI, Genomic and Immunotherapy Medical Institute, Université Bourgogne Franche‐Comté Dijon France; ^7^ INSERM, CIC1432, Module Epidémiologie Clinique, Dijon, France; Centre Hospitalier Universitaire Dijon‐Bourgogne, Centre d'Investigation Clinique, module Epidémiologie clinique/essais cliniques Dijon France; ^8^ Unité de Biologie Moléculaire Centre de Lutte Contre le Cancer Georges François Leclerc—UNICANCER Dijon France; ^9^ Institut de Chimie Moléculaire de l'Université de Bourgogne, UMR CNRS 6302 Dijon France

**Keywords:** genetic counseling, incidental findings, information, oncogenetic, theranostic exome sequencing

## Abstract

**Introduction:**

With the emergence of targeted therapies, there is a need to accurately identify more tumor biomarkers. The EXOMA trial was designed to offer tumor and germline exome sequencing (ES) to patients with solid malignant tumors and facing therapeutic failure. As hereditary cancer predispositions could be identified, with genetic counseling and health management implications, a genetic consultation was systematically established. This design needs to be discussed as genetic human resources are limited and indication of theranostic tests will increase.

**Methods:**

Genetic counseling was conducted within 15 days following inclusion in the study for patients recruited between December 2015 and July 2019. *In silico* analyses from theranostic ES were limited to 317 genes involved in oncogenesis, from both tumor and blood DNA.

**Results:**

Six hundred and sixty six patients had a genetic consultation before ES. In 65/666 patients, 66 germline pathogenic or likely pathogenic (P/LP) variants were identified in 16 actionable genes and seven non‐actionable genes according to French guidelines. 24/65 patients had previously received genetic analysis for diagnostic purposes, and for 17 of them, a P/LP variant had already been identified. Among the 48/65 remaining cases for which the EXOMA protocol revealed a previously unknown P/LP variant, only 19 met the criteria for genetic testing for inherited cancer risk after familial survey. These criteria had not been identified by the oncologist in 10 cases. In 21/65 cases, the variant was considered incidental.

**Discussion:**

In 7.4% of patients, an undiagnosed hereditary genetic predisposition was identified, whether or not related to the clinical presentation, and germline analysis impacted oncological management for only 6.3% of the cohort. This low percentage should be weighed against the burden of systematic genetic consultation and urgent circuits. Information or training tools to form oncologists to the prescription of germline genetic analyses should be explored, as well as information supports and patient preferences.

## 1 INTRODUCTION

The medical management of patients with solid malignancies has significantly changed in recent years. It is now known that cancers occur secondary to the appearance of genetic variants within tumor cells and that each tumor has its own genetic profile. The improvement of sequencing techniques has led to a better understanding of the molecular mechanisms involved in oncogenesis and has made it possible to identify new therapeutic targets. From nonspecific treatment with numerous adverse effects, we have observed the emergence of new targeted therapies that require detailed knowledge of the genetic characteristics of each tumor.^1^, ^2^, ^3^, ^4^

The EXOMA study, conducted between May 2016 and October 2018, was developed to provide systematic sequencing of the tumor and germline exome in patients with metastatic or locally advanced solid malignancies facing therapeutic failure, and to evaluate the feasibility and clinical utility of this approach.^5^ Genetic testing was performed not only for diagnostic purposes, but as theranostic analysis in order to propose appropriate targeted therapies following the identification of genetic biomarkers. We found, through this study, that exome sequencing (ES) was feasible in routine cancer care. This strategy enhanced access to targeted therapies and improved the detection of hereditary genetic predispositions. However, it also revealed unsuspected genetic susceptibilities to cancer, whether linked or not to the patient's phenotype.

In an effort to appropriately manage the identification of predispositions to cancer and to frame the germline analysis of this study, a systematic consultation with a geneticist or a genetic counsellor was implemented during the EXOMA study. This consultation, which was considered urgent and scheduled accordingly, aimed to provide patients with details on the implications of a germline genetic analysis. It could be difficult for oncologists to deliver this detailed information because of the time required. Moreover, some germline results could be complex to manage by non‐geneticist physicians. Considering that large genetic analyses for theranostic purposes will likely become increasingly common, we aimed to discuss the applicability of this theranostic circuit with a systematic and urgent genetic consultation in routine. In this ancillary study of the EXOMA trial, we discuss the implementation of these genetic consultations and their diagnostic accuracy with regard to the number of variants identified and the genes involved, especially in the absence of personal and/or familial criteria that would typically justify a genetic analysis.

## 2 METHODS

The EXOMA trial (NCT02840604) was a multicenter, prospective clinical trial, for which detailed methods and results were previously described.^5^ Eligible patients presented with metastatic cancer progressing after at least one line of systemic therapy. The main tumor type was breast cancer (21.5%), followed by colorectal (14.8%), and pancreatic cancer (14.2%) which reflected the classical recruitment of metastatic patients in including centers. Genetic testing for inherited cancer risk required a genetic consultation. Our current ancillary study was conducted in parallel with the EXOMA trial. We included all patients who had genetic counseling in the context of the EXOMA study and over a wider period, from December 2015 to July 2019, and who met the inclusion criteria in the EXOMA trial.

Tumor and germline ES with an *in silico* approach for analyzing 317 genes implicated in oncogenesis were proposed (Table S1). The EXOMA study solely focused on the impact of tumor and germline genetic variants on the oncological management of patients. An ES‐based approach will allow sequencing raw data to be reanalyzed in line with the scientific advances and the identification of new genes associated with an increased hereditary cancer risk. Non‐cancer related genes, as defined by the American College of Medical Genetics (ACMG) Secondary Findings v1,^6^ could not be analyzed according to the French legislation. Copy number variants were not analyzed. All methodology details on exome capture, sequencing, and analysis pipeline for tumor and germline variants were previously described.^5^

Median time from sample reception to delivery of the results was 52 days. Variants were classified according to the ACMG guidelines.^7^ Sanger sequencing confirmation was systematically performed for single nucleotide variant (SNV) and short indels classified as pathogenic or likely pathogenic (P/LP). All variants identified in germline ES were confirmed in the tumor analysis. Two classifications were used to discuss our results. First, genes were defined as actionable (genes for which there are prophylactic, diagnostic or therapeutic measures if a P/LP variant is identified) or non‐actionable (all other cases), according to the current French recommendations of the *Groupe Génétique et Cancer* ‐ UNICANCER (see URL GGC). Second, variants were classified into two groups: high/moderate penetrance (defined as cancer onset occurring respectively in >30% or 17%–30% of patients respectively) and low penetrance (<17%) or insufficient data (when literature data does not allow to establish a significant association between variant carriage and cancer occurrence), according to the ACMG, National Comprehensive Cancer Network (NCCN), and American Society of Clinical Oncology (see URLs ASCO and NCCN).

**FIGURE 1:**
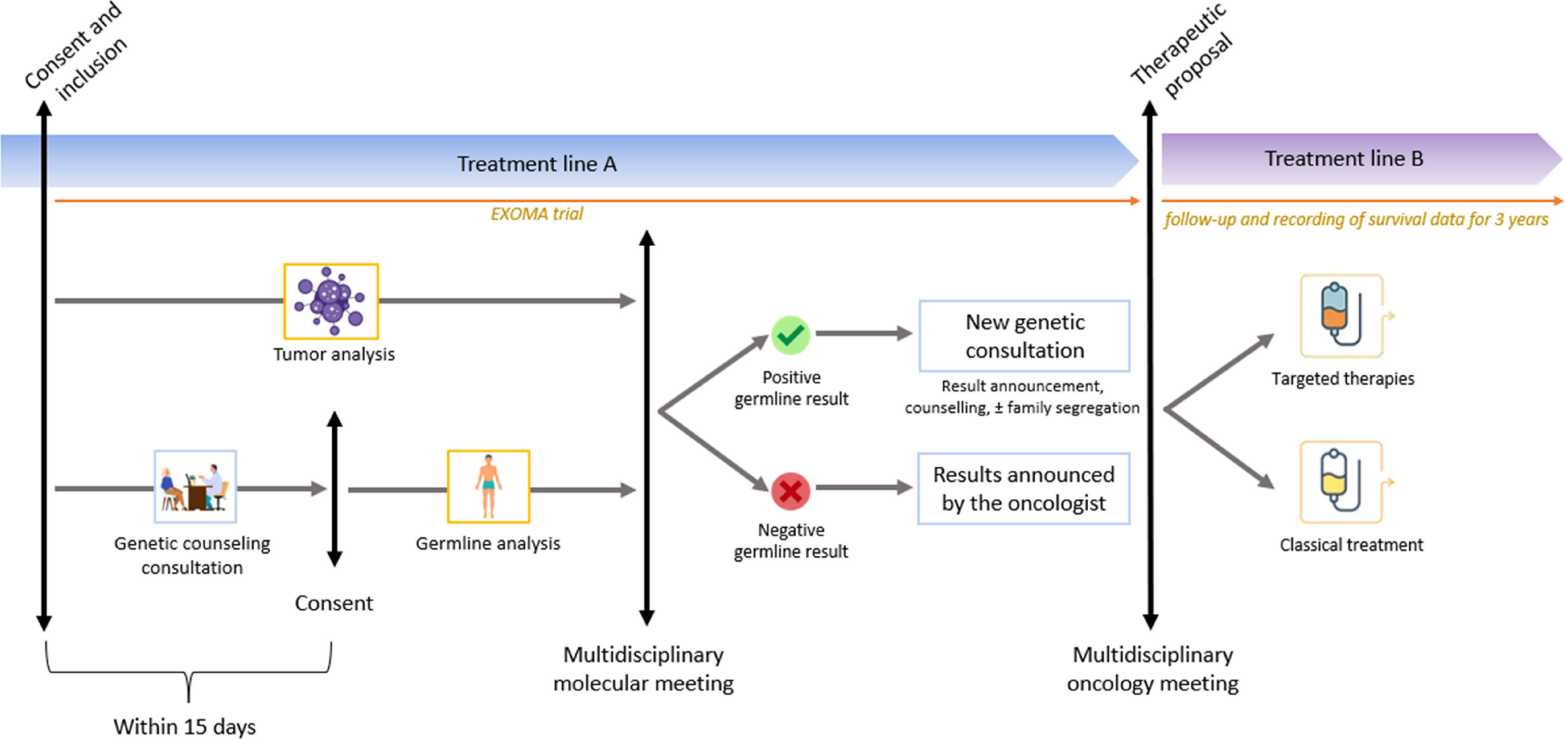
EXOMA study design and procedure.

During the EXOMA trial, each patient met with a geneticist or genetic counsellor for an in‐person genetic consultation within 15 days of inclusion in the study (Figure 1). Consultations lasted approximately 15–20 min and were informed by the prior medical history transmitted by the patient. Patients were seen again in person for a genetic consultation for explanation on the results if a hereditary P/LP variant or variant of unknown significance (VUS) was detected. If no P/LP variant or VUS was detected, the results were returned by the oncologist. A personalized management program for the patient with germline P/LP variant was discussed and defined in a multidisciplinary meeting according to the identified hereditary cancer risk(s). Presymptomatic testing was proposed to relatives of the patients for genes considered to be actionable in France. Retrospectively, the medical records of the patients for whom a germline variant was found were analyzed from our OncoGene database. In each case, we checked whether patients presented the criteria for hereditary cancer testing, and whether genetic testing or counseling had already been ordered/identified/performed because of personal and/or family history of cancer that would justify genetic testing. Indication criteria for genetic testing for cancer risk were defined according to the French recommendations of the *Groupe Génétique et Cancer* in effect at the time of inclusion.

In this ancillary study, only P/LP germline variants and VUS (ACMG class 3, 4, and 5)^6^, ^7^ identified in the analyzed genes were reported. The results of actionable genes with an impact on the patient were dissociated from the results of non‐actionable genes, according to French recommendations. Heterozygous *MUTYH* or *NTHL1* variants are mentioned in the results because the patients had a second genetic consultation for delivery of the results and to verify the family history. However, these cases were not included in the tally of the clinically relevant P/LP variants given the autosomal recessive nature of the genetic predisposition.

## 3 RESULTS

From December 2015 to July 2019, we included 666 patients undergoing tumor and germline ES for theranostic purposes and who attended a genetic consultation in the 15 days following inclusion.

**FIGURE 2:**
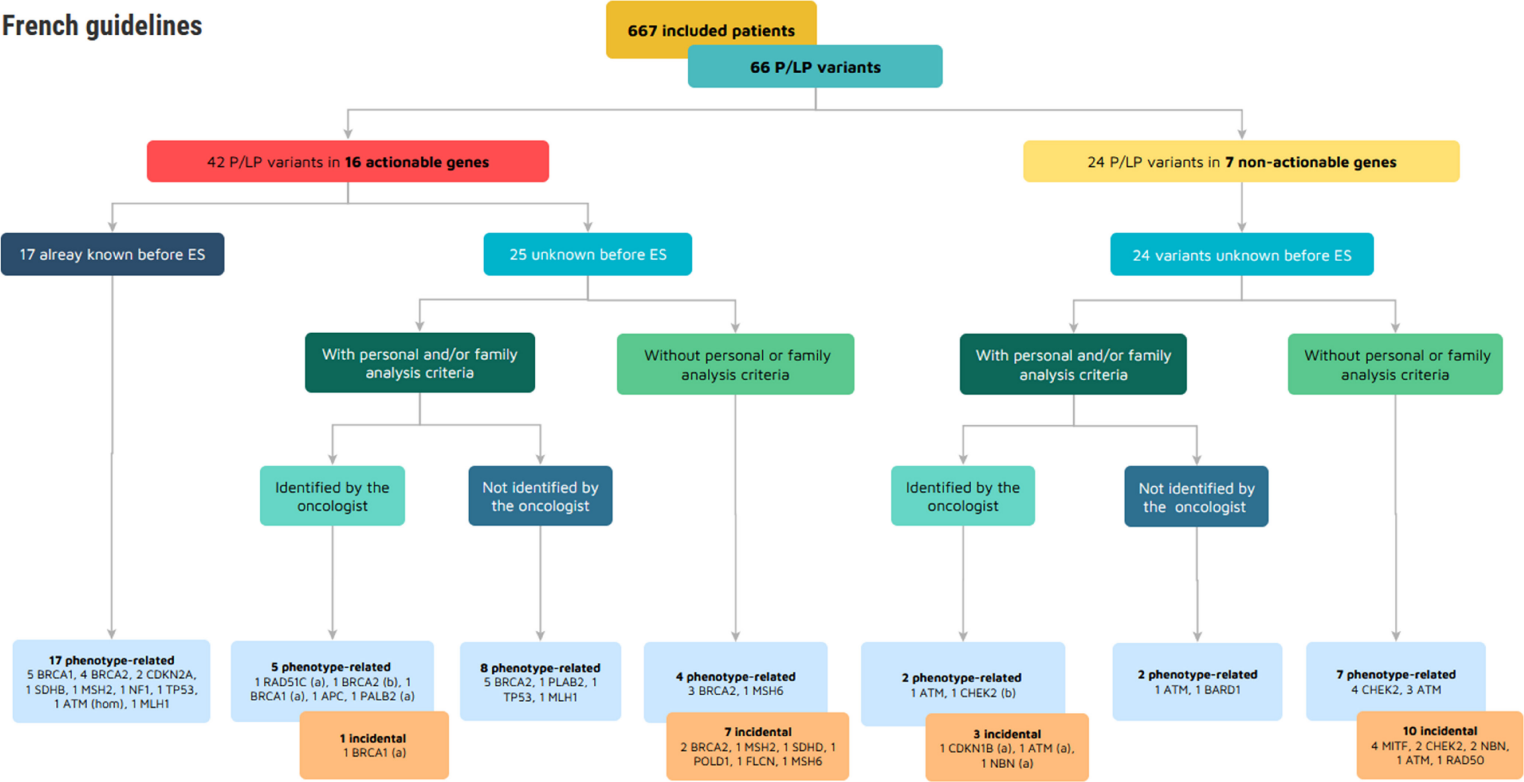
Genes in which P/LP variants were identified, distributed according to their identification before ES, the presence of analysis criteria and their identification by the oncologist, and variants causality defined according to the French recommendations (GGC). ES, exome sequencing; *n*, number of patients; P/LP, pathogenic or likely pathogenic. (a) Variant identified after a previous negative genetic analysis, (b) same patient, carrying two P/LP variants.

In 65/666 patients (9.8% of the cohort), 66 germline P/LP variants were identified. 42/66 variants (63.6%) were identified in 42 patients (6.3% of the whole cohort) within 16 actionable genes (mainly in *BRCA2* and *BRCA1*) (Table 1 and Figure 2). In 24 patients (3.6% of the whole cohort), 24/66 variants (36.4%) were identified within 7 genes that are not yet considered as actionable in France: *CHEK2*, *MITF*, *RAD50*, *CDKN1B*, *BARD1*, *NBN,* and heterozygous variants of *ATM*. One patient was counted twice because he carried two P/LP variants: one in *BRCA2* (actionable, c.7180A > T; p.Arg2394*), and one in *CHEK2* (non‐actionable, c.697G > T; p.Glu233*). All the identified variants are reported in Table S2.

**TABLE 1:** Main results observed in our study.

Genes	Number of variants (% of all P/LP variants identified)	Number of variants already known before EXOMA (% if relevant)	Number of variants with pre‐existing personal and/or family criteria for genetic analysis, phenotype‐related or not (% if relevant)	Number of variants with analysis criteria not identified by the oncologist (% if relevant)	Number of incidental variants (% if relevant)
Actionable genes					
*BRCA1*	7 (8.6)	5/7 (71.4)	7/7	0/7	1/7 (14.3)
*BRCA2*	15 (18.5)	4/15 (26.7)	9/15 (60.0)	5/9 (55.6)	2/15 (13.3)
*PALB2*	2 (2.5)	0/2	2/2	1/2	0/2
*RAD51C*	1 (1.2)	0/1	1/1	0/1	0/1
*MLH1*	2 (2.5)	1/2	2/2	1/2	0/2
*MSH2*	2 (2.5)	1/2	1/2	0/1	1/2
*MSH6*	2 (2.5)	0/2	0/2	N/A	1/2
*APC*	1 (1.2)	0/1	1/1	0/1	0/1
*POLD1*	1 (1.2)	0/1	0/1	N/A	1/1
*FLCN*	1 (1.2)	0/1	0/1	N/A	1/1
*NF1*	1 (1.2)	1/1	1/1	0/1	0/1
*TP53*	2 (2.5)	1/2	2/2	1/2	0/2
*CDKN2A*	2 (2.5)	2/2	2/2	0/2	0/2
*SDHB*	1 (1.2)	1/1	1/1	0/1	0/1
*SDHD*	1 (1.2)	0/1	0/1	N/A	1/1
*ATM* (hom)	1 (1.2)	1/1	1/1	0/1	0/1
Total	42/81 (51.9)	17/42 (40.5)	30/42 (71.4)	8/30 (26.7)	8/42 (19.0)
Non‐actionable genes					
*CHEK2*	7 (8.6)	0/7	1/7 (14.3)	0/1	2/7 (28.6)
*ATM* (het)	7 (8.6)	1/7 (14.3)	3/7 (42.9)	1/3 (33.3)	2/7 (28.6)
*BARD1*	1 (1.2)	0/1	1/1	1/1	0/1
*RAD50*	1 (1.2)	0/1	0/1	N/A	1/1
*MITF*	4 (4.9)	0/4	0/4	N/A	4/4
*NBN*	3 (3.7)	0/3	1/3 (33.3)	0/1	3/3
*CDKN1B*	1 (1.2)	0/1	1/1	0/1	1/1
Total	66/81 (81.5)	18/66 (27.3)	37/66 (56.1)	10/37 (27.0)	21/66 (31.8)
Others (heterozygous variants of recessive conditions)					
*MUTYH*	13 (16.0)	1/13 (7.7)	7/13 (53.8)	N/A	N/A
*NTHL1*	2 (2.5)	0/2	0/2	N/A	N/A
Total	81/81 (100)	19/81 (23.5)	44/81 (54.3)	10/44 (22.7)	21/81 (26.0)

Abbreviations: het, heterozygous; hom, homozygous; N/A, not available.

Of the 65 patients with a germline variant, 24 (36.9% and 3.6% of the whole cohort) were already identified in the care circuit and had benefited from at least one genetic germline analysis (targeted test or panel gene sequencing) due to the presence of personal and/or family criteria. For 17/24 of them (70.8% and 2.6% of the whole cohort), a P/LP variant had been identified before ES.

Of the 48 remaining patients (7.2% of the whole cohort) who had not had any genetic testing before the EXOMA trial, 25 P/LP variants were identified in actionable genes in 25 patients (3.8% of the whole cohort). The variant was phenotype‐related for only 17 of them (2.6% of the whole cohort). Among the 48 patients carrying a P/LP variant detected by ES, 34/48 (70.8% and 5.1% of the whole cohort) did not meet the individual criteria for genetic testing for hereditary cancer risk. However, 6/34 (17.6%) met the familial criteria for genetic predisposition analysis. In total, 28 patients (4.2% of the total cohort) did not present the individual or familial criteria for genetic testing for hereditary cancer risk, and 20 patients (3.0% of the cohort) met these criteria for analysis. In 10 of these 20 cases (1.5% of the whole cohort), these criteria had not been identified by the oncologist, and the patient had not been referred to a genetics specialist.

In 21/666 cases (3.2% of the cohort), the variant could be considered incidental since it was unrelated to personal or familial phenotypes (Table 2). Among these 21 patients, eight variants were identified in actionable genes and 13 in non‐actionable genes. Incidental findings are presented in Table 2.

**TABLE 2:** Incidental findings observed in our study.

Patient's cancer location (age of onset)	Family history of cancer (age of onset)	Genes	Variants identified	
Actionable genes				
Thyroid (57)	Thyroid and gynecologic	*BRCA1*	c.3839_3841delCTC	p.Ser1280_Gln1281delins*
Cervix (42)	Sister with breast cancer (42)	*BRCA2*	c.2612C > A	p.Ser871*
Lung (64)	–	*MSH6*	c.4075_4076insAATT	p.Ter1361Ilefs*4
Cervix (46)	–	*SDHD*	c.149A > G	p.His50Arg
Lung (55)	Pancreas, prostate, uterine cancer	*BRCA2*	c.5345_5346delAA	p.Asn1784Hisfs*2
Bone (62)	–	*MSH2*	c.32 T > A	p.Leu11*
Cervix (39) oesophagus (62)	–	*POLD1*	c.2087delG	p.Ser696Thrfs*32
Pancreas (73)	–	*FLCN*	c.553 T > C	p.Ser185Pro
Non‐actionable genes				
Breast (50)	–	*RAD50*	c.1875C > G	p.Tyr625*
Pancreas (70)	–	*MITF*	c.1255G > A	p.Glu419Lys
Breast (60)	–	*MITF*	c.1255G > A	p.Glu419Lys
Cervix (80) colon (80)	Sister with breast (71) and colon cancer (84)	*NBN*	c.37 + 1G > A	p.?
Bladder (54)	Breast/ovary syndrome	*MITF*	c.1255G > A	p.Glu419Lys
Endometrium (32)	Prostate and breast/ovary syndrome	*CHEK2*	c.1298A > C	p.Tyr433Ser
Ovary (58)	–	*NBN*	c.1909‐1910AT > TA	p.Ile637*
Pancreas (61)	Brother with kidney cancer	*MITF*	c.1255G > A	p.Glu419Lys
Pancreas (71)	–	*CHEK2*	c.720DelA	p.Val241Phefs*7
Pancreas (67)	–	*NBN*	c.657_661delACAAA	p.Lys219Asnfs*19
Ovary (28)	–	*CDKN1B*	c.206C > T	p.Pro69Leu
Biliary tract (64)	Lynch syndrome (*MSH2*), patient not carrier of the familial mutation	*ATM*	c.2921 + 1G > A	p.?
Lung (53)	Breast/ovary syndrome	*ATM*	c.1229 T > C	p.Val410Ala

**FIGURE 3:**
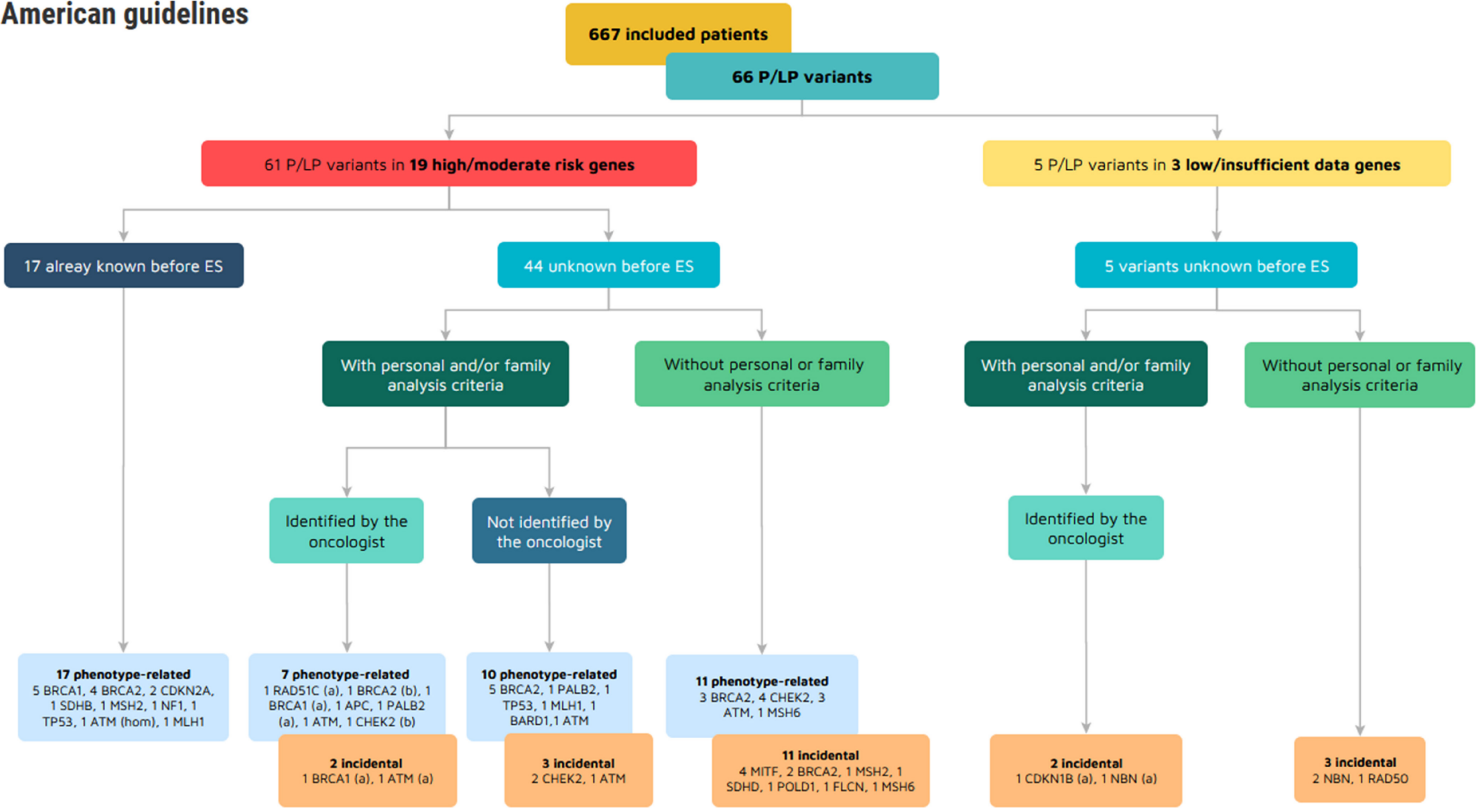
Genes in which P/LP variants were identified, distributed according to their identification before ES, the presence of analysis criteria and their identification by the oncologist, and risk level of variants defined according to the American guidelines. ES, exome sequencing; *n*, number of patients; P/LP, pathogenic or likely pathogenic. (a) variant identified after a previous negative genetic analysis, (b) same patient, carrying two P/LP variants.

Following the American guidelines and the classification of genes according to the risk level of cancer, 61/66 variants were identified in 19 moderate/high risk genes, and 5/66 in 3 low penetrance/insufficient data genes (Figure 3). The differences observed in our findings are related to *MITF*, *BARD1*, *CHEK2*, and *ATM* heterozygous variants. According to the NCCN guidelines, they are linked to a moderate risk of cancer development, which prompts preventive measures for carriers, and familial testing. In France, these variants are categorized as non‐actionable with no specific measures of surveillance nor familial testing recommended because of lacking/discordant literature data or insufficient risk.

We also identified 13 *MUTYH* heterozygous P/LP variants in 13 patients (2.0% of the cohort) and 2 *NTHL1* heterozygous P/LP variants in two patients (0.3% of the cohort). These alterations were not taken into account in the total number of P/LP variants mentioned above because of the lack of associated clinical impact.

Finally, 173 germline variants of unknown significance (VUS) were identified in 146 patients (22% of the cohort): 19 *POLE*, 17 *APC*, 14 *MSH6*, 14 *PMS2*, 12 *BRCA2*, 12 *SDHD*, 11 *PALB2*, 7 *RET*, 7 *CDH1*, 6 *BRCA1*, 6 *POLD*, 6 *MET*, 5 *MSH2*, 4 *MLH1*, 4 *MUTYH*, 4 *FLCN*, 3 *SMAD4*, 3 *MITF*, 2 *SDHB*, 2 *RAD51D*, 2 *BAP1*, 2 *DICER1*, 2 *CDKN2A*, 1 *ATM*, 1 *BMPR1A*, 1 *EPCAM*, 1 *NF1*, 1 *PTEN*, 1 *RAD51C*, 1 *RB1*, 1 *SDHC*, 1 *MEN1*. These results were returned to the patients in a second genetic consultation but did not modify their oncological management or family recommendations. However, patients were given the chance to request a reanalysis of the data, at least 2 years after the return, either at their own initiative or at the recommendation of their oncologist. Results of any reanalysis were not collected in this study, as they were not part of the protocol.

## 4 DISCUSSION

The advent of genomic medicine and the increasing use of high‐throughput sequencing in cancer patients has led to the discovery of new treatment targets and the emergence of personalized medicine. However, the use of increasingly broad sequencing techniques (multigene panels, ES, genome sequencing) is also forcing physicians to deal with an ever‐increasing amount of data: more tumor data could potentially be inherited, with wider implications for the management of patients and their families, and more incidental data can arise.^8^, ^9^, ^10^ While various recommendations have been published to help report incidental and secondary germline findings,^6^, ^11^, ^12^, ^13^ the discovery of such data still raises a number of ethical issues (such as management of incidental data, incomplete penetrance, variable expressivity, information for the family, insurance, etc.). Because of these issues, a specific care pathway requiring the assistance of a geneticist/genetic counselor is sometimes—but not systematically—set up by oncology teams before genetic testing for hereditary cancer risk. This approach is applicable before germline analysis as well as tumor testing since tumor variants can be inherited.

While the benefits of providing patients with such information before a genomic sequencing have already been studied,^10^, ^14^, ^15^ the place of a pretest genetic consultation within this emerging field of personalized medicine, relying on theranostic analysis, should be discussed. For the EXOMA trial, which was the first theranostic study including tumor and germline ES in our center, we chose to implement a systematic genetic consultation for the patient shortly after inclusion. We discuss here the place of this consultation in the care pathway and its contribution in the oncological management of patients with solid cancer experiencing therapeutic failure. We discuss the numbers of pathogenic/likely pathogenic variants, including the incidental findings, and then the feasibility of maintaining such circuits confronted with the number of concerned patients.

Through our study design, which involved a collaborative effort between the oncology and genetics teams and encompassed a systematic tumor and germline ES, we were able to influence oncological management (whether in terms of treatment, monitoring and/or screening of relatives) for three distinct categories of patients: (1) those who had a personal or familial indication for genetic analyses that had not been identified by their oncologist and for whom a causal gene was found (10/666 patients; 1.5%), (2) those for whom there was no personal or familial indication but for whom the analysis identified a causal gene (11/666 patients; 1.7%), and (3) those for whom incidental data was identified (regardless of whether or not there was an indication to perform the analysis) and who consented to be informed (21/666 patients; 3.2%). Patients carrying a P/LP causative variant and with a personal or familial indication identified by the oncologist would have been referred to a geneticist anyway and we believe that our study design did not provide a diagnostic benefit for them. Therefore, oncological management was impacted for 42 patients (6.3% of the cohort) and was not modified for the rest of the cohort (624 patients, 93.7%). These findings have certain limitations as they fail to consider potential future variants that might be discovered through reanalyzing the exome data. Additionally, they do not account for the recommendations given to patients regarding the surveillance of their relatives even if no pathogenic variant has been identified. Nevertheless, our results suggest that oncological management was impacted in only a small proportion of patients.

During the EXOMA study, 49 P/LP variants (excluding heterozygous *MUTYH* and *NTHL1*) were identified for the first time in 48 patients (7.2% of the whole cohort), in actionable and non‐actionable genes. 28/49 variants were considered to be linked to the patient phenotype.

It should be noted, however, that the definition of “actionable genes”, in the absence of international consensus, may vary between countries and may skew our results to underestimate the impact of germline findings. For example, the management of patients carrying *MUTYH* P/LP heterozygous variants has long been unclear, and the benefit/risk balance of systematic screening of such patients has only been recently determined in France.^16^ We chose to present our results based on a dual classification approach, incorporating the French guidelines, and the American recommendations to ensure a comprehensive analysis of the identified genetic variants. By utilizing the French classification, which focuses on the actionable/non‐actionable character of genes, we aim to emphasize its relevance in guiding clinical decision‐making, and treatment options. As our research was conducted within a French medical context, the French guidelines hold significant importance in management, and therapeutic strategies employed by professionals in local healthcare system. However, the American model of gene classification based on penetrance and risk level offers significant advantages and provides a more comprehensive understanding of the genetic landscape, allowing for a more nuanced evaluation of the clinical significance of these variants. One of the key advantages of the American model lies in its ability to identify genetic variants associated with moderate penetrance, such as those found in genes like *MITF*, *BARD1*, *CHEK2*, and *ATM*. Although classified as non‐actionable in France, the American approach recognizes that they still confer a notable risk of cancer onset, albeit at a moderate level. This crucial distinction allows for the implementation of preventive measures in carriers, empowering healthcare professionals to adopt targeted surveillance.

21/49 variants were considered to be incidental. Our results on the percentage of incidental data are consistent with previous studies.^8^, ^10^, ^17^ Numerous studies support the systematic use of large genetic studies, such as multigene panels, in oncology,^18^, ^19^, ^20^ particularly for the benefits in terms of patient diagnosis and survival, and family prevention. Yet, few studies have attempted to assess how patients feel about the discovery of incidental data with such techniques. In 2021, Nambot et al. showed that most of a small cohort of patients who received an incidental finding (IF) after genetic analysis for inherited cancer risk for which testing was indicated reported no major psychological burden, and almost all patients correctly initiated the recommended follow‐up. However, this work highlights the importance of receiving appropriate pretest information.^14^ Carrasco et al., 2022, provided some clarification regarding the psychological impact of genetics findings for cancer predisposition, including whether the patient had a family history of cancer or not. In a cohort of 533 individuals for whom ES was ordered for non‐oncological diagnosis, the authors identified secondary or incidental findings (SF/IF) in 2.1% of patients. They showed that, though the psychological impact of SF/IF disclosure was not clinically relevant, the numerical psychological impact scores were significantly higher in individuals receiving SF/IF than in individuals with a family history of cancer receiving similar genetic results.^15^ In the field of rare diseases, Gereis et al. (2022) indicated that ES or genome sequencing could be challenging for families to understand and underscored the importance of equipping healthcare professionals to explore parents' understanding of ES/genome sequencing and the implications of testing for their child.^21^ Houdayer et al. (2019) conducted focus groups with parents of patients with undiagnosed rare diseases potentially eligible for ES and patients affected by genetic predisposition to cancer, cardiogenetics or metabolic diseases. The researchers underscored the four key information‐based issues for participants, which ranked as follows: explanation of SF issues, autonomy of choice, importance of a reflection period, and quality of interactions between patients and professionals.^22^ Furthermore, Thauvin‐Robinet et al. (2019) highlighted the fact that the impact of the additional economic burden for deploying the search for SF/IF on organization of care should not be neglected.^23^ Further tumoral analyses and in‐depth family studies will be carried out to see whether the incidental nature of these variants can be maintained.

Considering our results and the low proportion of patients whose oncological management was impacted by genetic analysis, it seems legitimate to question the systematic use of a pretest genetic consultation. In the EXOMA study, exome analysis was negative for 587 patients (including 362 patients with no individual or familial criteria) and did not provide additional information for another 17 patients for whom a previous analysis had provided the same results as ES. Germline analysis impacted oncological management for only 6.3% of the cohort and among them 10 patients (1.5% of the cohort) had a personal or familial indication for genetic testing that had not been identified by their treating physician and could have been referred to a geneticist earlier. We believe that these misidentifications could be linked to two factors: a lack of familiarity with these criteria, and the lack of time available for oncology teams to collect and analyze patients' family histories.

Given the scarcity of genetics professionals, our approach of a systematic pretest genetic consultation is likely impractical and must be balanced against the organizational, economic, human and material resources that such an organization requires. However, full and clear upstream information must not be sacrificed because of a lack of human resources. Indeed, we do know that good communication and understanding of information increases adherence and compliance with the care process.^24^ In addition, more and more patients have expressed a desire to be actively involved in decision making about further treatment.^25^ Therefore, if the implementation of a systematic pretest genetic consultation is not sustainable, alternative information strategies need to be considered. Several options could help oncologists unfamiliar with genetic analyses to provide the necessary information before prescribing them: training can be offered by the in‐house genetics team (including the principle and regulatory aspects of the prescription) and standardized information can be transmitted during the consultation by means of a leaflet, small booklet, or a short video containing all the explanations about genetic testing for hereditary cancer risk and incidental data. Our team has the project to study the patients' preferences concerning these different means of information and to assess and compare their psychological impact and benefits.

In conclusion, our study found that it is difficult to recommend a systematic genetic consultation before ES since it led to a modified oncological management for only 6.3% of patients. Considering that large genomic analyses tend to be generalized in the management of cancers, and that French human resources in genetics are limited, it seems preferable to develop alternative approaches to patient information and to broaden the education of oncologists and society at large. Nevertheless, the role of the geneticist, as part of the multidisciplinary team, remains indispensable during these genetic analyses and for the return of a positive result. As Pujol et al. (2019) emphasize, the identification of a pathogenic variant in a known predisposing gene during a tumor analysis (and, *a fortiori*, during a germline analysis) should lead in a referral to a geneticist. Similarly, at any time during the analysis process, the patient should have access to a consultation with a geneticist if additional information is requested.^26^

The EXOMA trial is continuing with a new study called EXOMA 2. In this study, no systematic pretest genetic consultation will be implemented. Pretest information will be provided by oncologists and the genetics team will only be consulted on request and to return positive germline results. Testing will include more advanced analyses of the DNA structure, such as number of mutations, deletion, loss of heterozygosity, amplification, fusion, presence of microsatellite instability, deficiency in homologous repair, and mutational tumor burden. Accordingly, the information that will be delivered to the patient will become more complex. The training of oncologists and the link with the genetics team remain, to this day, an essential part of the framework of a personalized multidisciplinary approach to patient management.

## 5 URLs

GGC: https://recherche.unicancer.fr/fr/les‐groupes‐d‐experts/groupe‐genetique‐et‐cancer/

ASCO: https://old‐prod.asco.org/practice‐patients/guidelines

NCCN: https://www.nccn.org/guidelines/category_2

## AUTHOR CONTRIBUTIONS

**Benoit Mazel:** Formal analysis (equal); writing – original draft (equal); writing – review and editing (equal). **Geoffrey Bertolone:** Data curation (equal); writing – original draft (equal). **Amandine Baurand:** Data curation (supporting). **Elodie Cosset:** Data curation (supporting). **Caroline Sawka:** Data curation (supporting). **Marion Robert:** Data curation (supporting). **Elodie Gautier:** Conceptualization (supporting); funding acquisition (supporting); methodology (supporting). **Allan Lançon:** Data curation (supporting); investigation (supporting); methodology (supporting). **Manon Réda:** Conceptualization (supporting); data curation (supporting); investigation (supporting); methodology (supporting). **Laure Favier:** Conceptualization (supporting); data curation (supporting); investigation (supporting); validation (supporting). **Valentin Dérangère:** Data curation (supporting); investigation (supporting). **Corentin Richard:** Data curation (supporting); investigation (supporting). **Christine Binquet:** Data curation (supporting); investigation (supporting). **Romain Boidot:** Data curation (supporting). **Vincent Goussot:** Data curation (supporting). **Juliette Albuisson:** Data curation (supporting). **François Ghiringhelli:** Conceptualization (equal); data curation (supporting); investigation (equal); methodology (equal). **Laurence Faivre:** Conceptualization (equal); data curation (supporting); investigation (equal); methodology (equal); supervision (equal); validation (equal); writing – review and editing (equal). **Sophie Nambot:** Data curation (equal); investigation (equal); validation (equal); writing – original draft (equal); writing – review and editing (equal).

## FUNDING INFORMATION

This work was funded by the grants from the Centre Georges François Leclerc and Dijon University Hospital, the ISITE‐BFC (PIA ANR) and the European Union through the FEDER programs. The funding sources had no role in the design and conduct of the study, collection, management, analysis and interpretation of the data, preparation, review or approval of the manuscript, or decision to submit the manuscript.

## CONFLICT OF INTEREST STATEMENT

The authors declare no conflict of interest.

## ETHICS STATEMENT

The Exoma trial (NCT02840604) was approved by the ethical review comitee called *Comité de Protection des Personnes Est*. Written informed consent was obtained from all individuals before participating in the study.

## Supporting information


**Supplementary TABLE 1.** List of genes analyzed by tumor and germline ES.
Supplementary Table 2. Variants identified and indication for genetic analysis.

## Data Availability

The data that support the findings of this study are available from the corresponding author, upon reasonable request.
