# Supplementary material for: Advancing precision oncology through systematic germline and tumor genetic analysis: The oncogenetic point of view on findings from a prospective multicenter clinical trial of 666 patients
Source: Cancer Med. 2023 Sep 11;12(18):18786–96. doi: 10.1002/cam4.6498 (PMC10557826; doi:10.1002/cam4.6498)
Supplement: Supplementary file 1 — Supplementary TABLE 1. List of genes analyzed by tumor and germline ES. Supplementary Table 2. Variants identified and indication for genetic analysis. [file CAM4-12-18786-s001.docx]

| *ABL1* | *ABL2* | *AKT1* | *AKT2* | *AKT3* | *ALK* | *AMER1* | *ANAPC2* | *APC* | *AR* |
| --- | --- | --- | --- | --- | --- | --- | --- | --- | --- |
| *ARAF* | *ARFRP1* | *ARID1A* | *ARID2* | *ASXL1* | *ATM* | *ATR* | *ATRX* | *AURKA* | *AURKB* |
| *AXIN1* | *AXIN2* | *AXL* | *BAP1* | *BARD1* | *BCL2* | *BCL6* | *BCOR* | *BCORL1* | *BLM* |
| *BRAF* | *BRCA1* | *BRCA2* | *BRIP1* | *BTK* | *BUB1B* | *C11orf30* | *CARD11* | *CBFB* | *CBL* |
| *CCND1* | *CCND2* | *CCND3* | *CCNE1* | *CD79A* | *CD79B* | *CDC25A* | *CDC34* | *CDC73* | *CDH1* |
| *CDK12* | *CDK4* | *CDK5RAP1* | *CDK6* | *CDK8* | *CDKN1B* | *CDKN2A* | *CDKN2C* | *CDKN3* | *CEBPA* |
| *CHEK1* | *CHEK2* | *CIC* | *CKIT* | *CREBBP* | *CRKL* | *CRLF2* | *CSF1R* | *CTCF* | *CTNNA1* |
| *CTNNB1* | *CUL1* | *CUL2* | *CUL3* | *DAXX* | *DDR2* | *DICER1* | *DNMT3A* | *DOT1L* | *DPYD* |
| *E2F1* | *EGFR* | *EP300* | *EPCAM* | *EPHA2* | *EPHA3* | *EPHA5* | *EPHB1* | *ERBB2* | *ERBB3* |
| *ERBB4* | *ERCC1* | *ERCC2* | *ERCC3* | *ERCC4* | *ERCC5* | *ERCC6* | *ERCC8* | *ERG* | *ESR1* |
| *ESR2* | *EZH2* | *FAM46C* | *FANCA* | *FANCC* | *FANCD2* | *FANCE* | *FANCF* | *FANCG* | *FANCL* |
| *FBXW7* | *FGF10* | *FGF14* | *FGF19* | *FGF23* | *FGF3* | *FGF4* | *FGF6* | *FGFR1* | *FGFR2* |
| *FGFR3* | *FGFR4* | *FLCN* | *FLT1* | *FLT3* | *FLT4* | *FOXL2* | *GATA1* | *GATA2* | *GATA3* |
| *GID4* | *GLI1* | *GLI2* | *GLI3* | *GNA11* | *GNA13* | *GNAQ* | *GNAS* | *GREM1* | *GRIN2A* |
| *GRP* | *GSK3A* | *GSK3B* | *HGF* | *HRAS* | *HSP90AA1* | *IDH1* | *IDH2* | *IGF1R* | *IKBKE* |
| *IKZF1* | *IL7R* | *INHBA* | *INPP4A* | *INPP4B* | *IRF4* | *IRS2* | *JAK1* | *JAK2* | *JAK3* |
| *JUN* | *KAT6A* | *KDM5A* | *KDM5C* | *KDM6A* | *KDR* | *KEAP1* | *KIT* | *KLHL6* | *KRAS* |
| *LCK* | *LRP1B* | *MAP2K1* | *MAP2K2* | *MAP2K3* | *MAP2K4* | *MAP3K1* | *MAPK1* | *MAPK3* | *MCL1* |
| *MDM2* | *MDM4* | *MED12* | *MEF2B* | *MEN1* | *MET* | *MITF* | *MLH1* | *MLH3* | *MPL* |
| *MSH2* | *MSH6* | *MTOR* | *MUTYH* | *MYC* | *MYCL1* | *MYCN* | *MYD88* | *NBN* | *NF1* |
| *NF2* | *NFE2L2* | *NFKBIA* | *NKX2-1* | *NLRP3* | *NOTCH1* | *NOTCH2* | *NPM1* | *NRAS* | *NTHL1* |
| *NTRK1* | *NTRK2* | *NTRK3* | *NUP93* | *PAK3* | *PALB2* | *PARP1* | *PARP2* | *PAX5* | *PBRM1* |
| *PDGFRA* | *PDGFRB* | *PDK1* | *PGR* | *PIK3CA* | *PIK3CG* | *PIK3R1* | *PIK3R2* | *PMS1* | *PMS2* |
| *POLD* | *POLE* | *PPP2R1A* | *PRDM1* | *PRKACA* | *PRKACB* | *PRKAR1A* | *PRKDC* | *PRSS1* | *PRUNE2* |
| *PTCH1* | *PTCH2* | *PTEN* | *PTPN11* | *RAD50* | *RAD51B* | *RAD51C* | *RAD51D* | *RAD54L* | *RAF1* |
| *RARA* | *RB1* | *RET* | *RICTOR* | *RNF43* | *ROS1* | *RPTOR* | *RUNX1* | *SDHAF2* | *SDHB* |
| *SDHC* | *SDHD* | *SETD2* | *SF3B1* | *SHH* | *SKP2* | *SLC28A1* | *SLC29A1* | *SMAD1* | *SMAD2* |
| *SMAD3* | *SMAD4* | *SMAD5* | *SMARCA4* | *SMARCB1* | *SMO* | *SOCS1* | *SOX10* | *SPARC* | *SPEN* |
| *SPOP* | *SRC* | *STAG2* | *STAT3* | *STAT4* | *STK11* | *SUFU* | *SUZ12* | *TERT* | *TET2* |
| *TGFBR2* | *THRA* | *THRB* | *TNFAIP3* | *TNFRSF14* | *TOP1* | *TP53* | *TP53BP1* | *TSC1* | *TSC2* |
| *TSHR* | *TYMS* | *UIMC1* | *VHL* | *WISP3* | *WNT* | *WT1* | *XPO1* | *XRCC1* | *XRCC2* |
| *XRCC3* | *XRCC4* | *XRCC5* | *XRCC6* | *YES1* | *ZNF217* | *ZNF70* |  |  |  |

**Supplementary data :**

Supplementary Table 1. List of genes analyzed by tumoral and germline exome sequencing.

| Genes | Patient’s cancer location(s) | Indication for genetic analysis (if present) | Variants identified | | Incidental finding |
| --- | --- | --- | --- | --- | --- |
| *APC* | Rectum | Digestive cancer | c.1938delA | p.Ile646Metfs*11 | No |
| *ATM* | Prostate and hematopoietic system | N/A | c.6115G>A | p.Glu2039Lys | No |
| *ATM (hom)* | Breast | Breast/ovarian syndrome | c.7271T>G (homozygous) | p.Val2424Gly (homozygous) | No |
| *ATM* | Pancreas and ovary | Breast/ovarian syndrome | c.8545C>T | p.Arg2849* | No |
| *ATM* | Breast | Breast/ovarian syndrome | c.1561_1562delAG | p.Glu552Ilefs*43 | No |
| *ATM* | Pancreas | N/A | c.8786+1G>A | p.? | No |
| *ATM* | Pancreas | N/A | c.331+2T>G | p.? | No |
| *ATM* | Biliary tract | Digestive cancer | c.2921+1G>A | p.? | Yes |
| *ATM* | Lung | N/A | c.1229T>C | p.Val410Ala | Yes |
| *BARD1* | Breast | Breast/ovarian syndrome | c.1690C>T | p.Gln564* | No |
| *BRCA1* | Ovary | Breast/ovarian syndrome | c.181T>G | p.Cys61Gly | No |
| *BRCA1* | Breast | Breast/ovarian syndrome | c.3937C>T | p.Gln1313* | No |
| *BRCA1* | Ovary | Breast/ovarian syndrome | c.1961delA | p.Lys654fs | No |
| *BRCA1* | Breast | Breast/ovarian syndrome | c.83_84delTG | p.Leu28Argfs*12 | No |
| *BRCA1* | Pancreas | Digestive cancer | c.3331_3334delCAAG | p.Gln1111Asnfs*5 | No |
| *BRCA1* | Breast | Breast/ovarian syndrome | c.1380dupA | p.Phe461Ilefs*19 | No |
| *BRCA1(a)* | Thyroid | Endocrine cancer | c.3839_3841delCTC | p.Ser1280_Gln1281delins* | Yes |
| *BRCA2* | Breast | Breast/ovarian syndrome | c.7654dupA | p.Ile2552Asnfs*2 | No |
| *BRCA2* | Ovary | Breast/ovarian syndrome | c.6209_6212delAAAG | p.Glu2070Valfs*10 | No |
| *BRCA2* | Uterine cervix | N/A | c.2612C>A | p.Ser871* | Yes |
| *BRCA2* | Breast | N/A | c.5851_5854DelAGTT | p.Ser1951Trpfs*11 | No |
| *BRCA2* | Breast | N/A | c.6079DupA | p.Arg2027Lysfs*22 | No |
| *BRCA2* | Breast | Breast/ovarian syndrome | c.9294C>A | p.Tyr3098* | No |
| *BRCA2* | Pancreas | Digestive cancer | c.9767_9770delAGAA | p.Argfs*17 | No |
| *BRCA2* | Pancreas | N/A | c.244A>T | p.Lys82* | No |
| *BRCA2* | Lung | N/A | c.5345_5346delAA | p.Asn1784Hisfs*2 | Yes |
| *BRCA2* | Pancreas | N/A | c.273C>A | p.Tyr91* | No |
| *BRCA2* | Pancreas and breast | Breast/ovarian syndrome | c.4284dupT | p.Gln1429Serfs*9 | No |
| *BRCA2* | Ovary and breast | Breast/ovarian syndrome | c.2539A>T | p.Arg847* | No |
| *BRCA2* | Breast | Breast/ovarian syndrome | c.244A>T | p.Lys82* | No |
| *BRCA2* | Pancreas | Digestive cancer | c.7558C>GT | p.Arg2520* | No |
| *BRCA2(b)* | Breast | Breast/ovarian syndrome | c.7180A>T | p.rg2394* | No |
| *CDKN1B* | Ovary | Breast/ovarian syndrome | c.206C>T | p.Pro69Leu | Yes |
| *CDKN2A* | Skin and colon | Melanoma and other skin tumors | c.176T>G | p.Val59Gly | No |
| *CDKN2A* | Skin and pancreas | Melanoma and other skin tumors | c.107delC | p.Ala36fs | No |
| *CHEK2(b)* | Breast | Breast/ovarian syndrome | c.697G>T | p.Glu233* | No |
| *CHEK2* | Breast | N/A | c.478A>G | p.Arg160Gly | No |
| *CHEK2* | Endometrium | N/A | c.1298A>C | p.Tyr433Ser | Yes |
| *CHEK2* | Breast | N/A | c.946_947delGA | p.Glu316Asnfs*16 | No |
| *CHEK2* | Pancreas | N/A | c.720DelA | p.Val241Phefs*7 | Yes |
| *CHEK2* | Breast | N/A | c.599T>C | p.Ile200Thr | No |
| *CHEK2* | Breast | N/A | c.1100delC | p.Thr367Metfs*15 | No |
| *FLCN* | Pancreas | N/A | c.553T>C | p.Ser185Pro | Yes |
| *MITF* | Pancreas | N/A | c.1255G>A | p.Glu419Lys | Yes |
| *MITF* | Breast | N/A | c.1255G>A | p.Glu419Lys | Yes |
| *MITF* | Bladder | N/A | c.1255G>A | p.Glu419Lys | Yes |
| *MITF* | Pancreas | N/A | c.1255G>A | p.Glu419Lys | Yes |
| *MLH1* | Biliary tract and skin | Digestive cancer | c.503dupA | p.Asn168fs | No |
| *MLH1* | Biliary tract, colon and endometrium | Digestive cancer | c.544A>G | p.Arg182Gly | No |
| *MSH2* | Prostate and rectum | Digestive cancer | c.2038C>T | p.Arg680* | No |
| *MSH2* | Bone | N/A | c.32T>A | p.Leu11* | Yes |
| *MSH6* | Lung | N/A | c.4075_4076insAATT | p.Ter1361Ilefs*4 | Yes |
| *MSH6* | Colon | N/A | c.2731C>T | p.Arg911* | No |
| *MUTYH(a)* | Thyroid | Endocrine neoplasia | c.1187G>A | p.Gly396Asp | N/A |
| *MUTYH* | Breast | Breast/ovarian syndrome | c.536A>G | p.Tyr179Cys | N/A |
| *MUTYH* | Lung | N/A | c.1187G>A | p.Gly396Asp | N/A |
| *MUTYH* | Colon | N/A | c.536A>G | p.Thy179Cys | N/A |
| *MUTYH* | Breast | Breast/ovarian syndrome | c.1187G>A | p.Gly396Asp | N/A |
| *MUTYH* | Connective tissue and prostate | N/A | c.1187G>A | p.Gly396Asp | N/A |
| *MUTYH* | Unknown | N/A | c.1187G>A | p.Gly396Asp | N/A |
| *MUTYH* | Breast | N/A | c.1187G>A | p.Gly396Asp | N/A |
| *MUTYH* | Pancreas | Digestive cancer | c.1187G>A | p.Gly396Asp | N/A |
| *MUTYH* | Ovary | Breast/ovarian syndrome | c.1227_1228dupGG | p.Glu410Glyfs*43 | N/A |
| *MUTYH* | Pancreas and breast | Breast/ovarian syndrome | c.1187G>A | p.Gly396Asp | N/A |
| *MUTYH* | Colon | N/A | c.536A>G | p.Thy179Cys | N/A |
| *MUTYH(c)* | Pancreas and breast | Breast/ovarian syndrome | c.1187G>A | p.Gly396Asp | N/A |
| *NBN* | Uterine cervix and colon | N/A | c.37+1G>A | p.? | Yes |
| *NBN* | Ovary | Breast/ovarian syndrome | c.1909-1910AT>TA | p.Ile637* | Yes |
| *NBN* | Pancreas | N/A | c.657_661delACAAA | p.Lys219Asnfs*19 | Yes |
| *NF1* | Peripheral nervous system | Peripheral nervous system tumor | c.1466a>G | p.Tyr489* | No |
| *NTHL1* | Stomach | N/A | c.268C>T | p.Gln90* | N/A |
| *NTHL1* | Lung and prostate | N/A | c.235dupG | p.Ala79Glyfs*2 | N/A |
| *PALB2* | Breast | Breast/ovarian syndrome | c.1407C>A | p.Cys469* | No |
| *PALB2(c)* | Pancreas and breast | Breast/ovarian syndrome | c.2257C>T | p.Arg753* | No |
| *POLD1* | Esophagus and uterine cervix | N/A | c.2087delG | p.Ser696Thrfs*32 | Yes |
| *RAD50* | Breast | N/A | c.1875C>G | p.Tyr625* | Yes |
| *RAD51C* | Ovary | Breast/ovarian syndrome | c.732delT | p.Ile244Metfs*9 | No |
| *SDHB* | Paraganglion | Endocrine neoplasia | c.93dup | p.Ala32Serfs*31 | No |
| *SDHD* | Uterine cervix | N/A | c.149A>G | p.His50Arg | Yes |
| *TP53* | Ovary | Breast/ovarian syndrome | c.844C>T | p.Arg282Trp | No |
| *TP53* | Muscle | Pediatric cancer | c.472C>T | p.Arg158Cys | No |

Supplementary Table 2. Variants identified and indication for genetic analysis. N/A: not available; hom: homozygous ; (a), (b), (c) : same patients carrying respectively two pathogenic or likely pathogenic variants.
